# Impacts of microbial assemblage and environmental conditions on the distribution of anatoxin-a producing cyanobacteria within a river network

**DOI:** 10.1038/s41396-019-0374-3

**Published:** 2019-02-26

**Authors:** Keith Bouma-Gregson, Matthew R. Olm, Alexander J. Probst, Karthik Anantharaman, Mary E. Power, Jillian F. Banfield

**Affiliations:** 1 Department of Integrative Biology, University of California, Berkeley, CA USA; 2 Department of Earth and Planetary Science, University of California, Berkeley, CA USA; 3 Department of Plant and Microbial Biology, University of California, Berkeley, CA USA; 4 Department of Environmental Science, Policy, and Management, University of California, Berkeley, CA USA; 5 Earth Sciences Division, Lawrence Berkeley National Laboratory, Berkeley, CA USA; 6 Chan Zuckerberg Biohub, San Francisco, CA USA; 7 Present Address: Group for Aquatic Microbial Ecology, Biofilm Center, Department for Chemistry, University of Duisburg-Essen, Essen, Germany; 8 Present Address: Department of Bacteriology, University of Wisconsin, Madison, WI USA

**Keywords:** Metagenomics, Microbial ecology, Freshwater ecology

## Abstract

Blooms of planktonic cyanobacteria have long been of concern in lakes, but more recently, harmful impacts of riverine benthic cyanobacterial mats been recognized. As yet, we know little about how various benthic cyanobacteria are distributed in river networks, or how environmental conditions or other associated microbes in their consortia affect their biosynthetic capacities. We performed metagenomic sequencing for 22 Oscillatoriales-dominated (Cyanobacteria) microbial mats collected across the Eel River network in Northern California and investigated factors associated with anatoxin-a producing cyanobacteria. All microbial communities were dominated by one or two cyanobacterial species, so the key mat metabolisms involve oxygenic photosynthesis and carbon oxidation. Only a few metabolisms fueled the growth of the mat communities, with little evidence for anaerobic metabolic pathways. We genomically defined four cyanobacterial species, all which shared <96% average nucleotide identity with reference Oscillatoriales genomes and are potentially novel species in the genus *Microcoleus*. One of the *Microcoleus* species contained the anatoxin-a biosynthesis genes, and we describe the first anatoxin-a gene cluster from the *Microcoleus* clade within Oscillatoriales. Occurrence of these four *Microcoleus* species in the watershed was correlated with total dissolved nitrogen and phosphorus concentrations, and the species that contains the anatoxin-a gene cluster was found in sites with higher nitrogen concentrations. Microbial assemblages in mat samples with the anatoxin-a gene cluster consistently had a lower abundance of Burkholderiales (Betaproteobacteria) species than did mats without the anatoxin-producing genes. The associations of water nutrient concentrations and certain co-occurring microbes with anatoxin-a producing *Microcoleus* motivate further exploration for their roles as potential controls on the distributions of toxigenic benthic cyanobacteria in river networks.

## Introduction

When cyanobacteria proliferate in freshwaters, their toxins can threaten water quality and public health [1]. Harmful cyanobacterial blooms in lakes have been described for decades (e.g. [2]). In rivers, however, first reports of animal deaths from toxic benthic cyanobacteria in many regions occurred only in the last 20 years [3–6]. Nevertheless, nuisance benthic cyanobacterial mats in rivers have been documented across the globe, including New Zealand [7, 8], California [9, 10], France [5], and Spain [11]. Benthic mats are often formed by filamentous genera such as *Anabaena*, *Phormidium, Microcoleus*, *Nodularia*, *Lyngbya*, or *Oscillatoria*, which sometimes produce cyanotoxins such as anatoxin-a, microcystins, saxitoxin, and lyngbyatoxin [3]. Cyanobacterial blooms in lakes and estuaries are predicted to increase due to eutrophication and climate warming [12, 13], and more study of riverine benthic cyanobacterial mats is needed to anticipate environmental and ecological triggers of toxigenic cyanobacterial blooms in rivers.

**Fig. 1:**
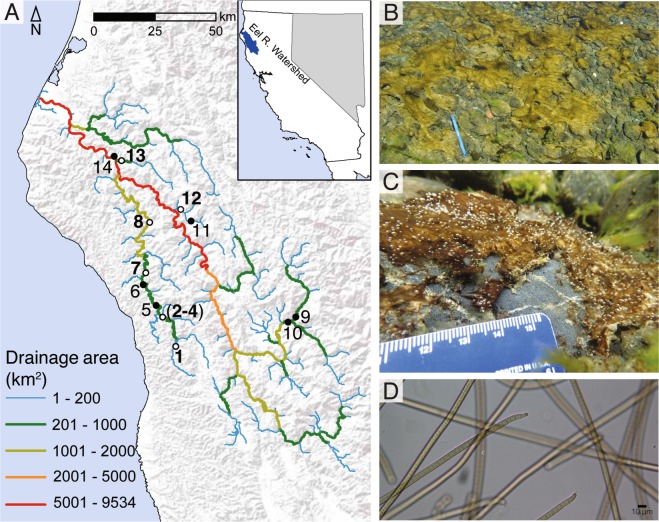
Locations of sampling sites and images of *Microcoleus*-dominated mats. **a** Map of Eel River watershed showing location of samples collected in the Eel River watershed in August 2015. At sites represented by white circles and bold numbers, upstream and downstream samples were collected (designated with U and D in the text), while a single sample was collected at sites represented by black circles (designated with S in the text). Sites 2–4 were in close proximity to one another at a tributary confluence and are represented by a single circle. **b** *Microcoleus*-dominated mats in the Eel River. **c** Underwater photograph showing *Microcoleus*-dominated mat on a cobble. **d** Micrograph of *Microcoleus* trichomes (400×)

Every summer in the Eel River in Northern California (Fig. 1), characteristic filamentous Oscillatoriales (Cyanobacteria) mats, magenta, maroon, green, or brown in color, appear as thin veils of prostrate, feathery filaments clinging to cobbles or boulders where river flow velocities exceed ~10 cm/s. The dominant Oscillatoriales taxa that form these mats have been morphologically identified as belonging to the cyanobacterial genus *Phormidium* [10]. The taxonomy of *Phormidium* has been complicated by several revisions [14–17], so different genus and species names have been applied to this group over time. Molecular phylogenies moved some *Phormidium* species to the genus *Microcoleus* [14–16], however, no molecular phylogenies have been constructed from Eel River *Phormidium* mats to determine if they belong to genus *Phormidium* or the genus *Microcoleus*.

Cyanobacteria are the foundational organisms that give rise to benthic mats and create habitat for other microbes to colonize. Heterotrophic bacteria often grow attached to cyanobacterial filaments or within cyanobacterial mats, exchanging nutrients and carbon [18, 19]. Both antagonistic [20, 21] and beneficial [18, 22] interactions between cyanobacteria and other microbes occur, although often rates of cyanobacterial growth [23] and other beneficial processes, such as nutrient exchange [24] and nitrogen fixation [25], are enhanced by co-occurring microbes. Characterizing the diversity and metabolisms of the whole microbial consortia within cyanobacterial mats should improve our understanding of these very local ecological controls over proliferation of toxigenic mats in rivers.

While much research has been performed on algal and microbial biofilms in rivers (e.g. [26–28]), we know of only one published study on microbial consortia of benthic toxigenic cyanobacteria in rivers, which documented shifts over time in the microbial assemblages of *Microcoleus* (formerly *Phormidium*, (Oscillatoriales)) mats in New Zealand [29]. Organisms in the phylum Proteobacteria were abundant in these assemblages, including taxa known to produce alkaline phosphatase, which may be important for mat growth in these phosphorus-limited New Zealand rivers [7, 30, 31]. The New Zealand mats were profiled using 16S rRNA gene amplicons surveys [29], a method that cannot provide specific information about metabolic functions or metabolites produced by microbes in the mats.

Cyanotoxins, especially anatoxin-a, have been detected in benthic cyanobacterial mats from the Eel River [4, 10]. Anatoxin-a is a neurotoxic alkaloid that inhibits neuromuscular nicotinic acetylcholine receptors by disrupting cellular ion channels, which causes muscle failure and can lead to death [32, 33]. To synthesize anatoxin-a, enzymes encoded in a 10-gene (*anaA-J*) polyketide synthase gene cluster transform proline, a cyclic amino acid, into anatoxin-a [34–36]. Anatoxin-a biosynthesis genes occur in distantly related cyanobacterial species, though some of the order of genes in the biosynthesis gene cluster is preserved among distantly related taxa [35, 37, 38]. Not all strains within species that produce anatoxin-a contain the anatoxin-a gene cluster, and variation between toxin and non-toxin producing strains occurs over small spatial scales (<1 cm) [39, 40]. The abundance of toxin-producing genotypes can drive changes in anatoxin-a concentrations within a mat, rather than differential toxin production per cell [40], a pattern common with other cyanotoxins as well [41].

Currently, there have been no culture-independent genome-resolved investigations of toxigenic freshwater cyanobacterial mats, which could provide high-resolution information about taxonomic diversity and insight into the metabolic potential of microbes living within the mats. We collected Oscillatoriales-dominated mats from 14 sites broadly distributed across the Eel River watershed and found the abundant Oscillatoriales taxa to belong to the genus *Microcoleus*, not *Phormidium*. Using genome-resolved metagenomics, we aimed to (1) characterize how the diversity of *Microcoleus* species changes along spatial and environmental gradients in the watershed; (2) assess the biosynthetic capacity for anatoxin-a by *Microcoleus* species and investigate links between the microbial assemblage composition and the potential for anatoxin-a production; and (3) understand how co-occurring microbes contribute to the flow of energy and cycling of nutrients through the mats.

## Materials and methods

### Sample collection

Samples were collected from *Microcoleus*-dominated mats over 3 weeks in August 2015 in the Eel River, a 9547 km^2^ watershed in the Northern California Coast Range (Fig. 1). The region has a Mediterranean climate with seasonal summer drought. The river is largely canyon-bound with little floodplain habitat. Relatively steep for its drainage area, the Eel’s bed substrates are dominated by pebbles, cobbles, and boulders. During the summer as river flow subsides and warms, attached filamentous algae proliferate, particularly in sunlit mainstems [42, 43]. Benthic cyanobacteria have poisoned dogs in the river [4], and anatoxin-a has been detected throughout the watershed [10].

The upstream drainage areas at sampling sites ranged from 17 km^2^ to 7908 km^2^. *Microcoleus*-dominated mats were identified macroscopically by their brown, orange, olive, or maroon coloration, and their characteristic epilithic growth. Microscopic identification confirmed that the mats were dominated by Oscillatoriales that were morphologically identified as *Phormidium* with a Nikon Optiphot II light microscope at ×400 using the key in [44] (Fig. 1). These mats were usually found in cobble and boulder bedded riffles. All samples were collected from water 5–70 cm deep with surface flows of 5–100 cm/s.

At each site, a cobble covered by a mat was lifted from the river and placed in a tray that had been cleaned with 70% ethanol. Using sterile forceps, ~0.5 g of biomass was sampled from the mat and placed in a sterile 2 mL cryotube (VWR microcentrifuge tube 89004–302). Samples were immediately flash-frozen by filling a cooler with 2 L of ethanol and then adding ~250 mL of dry ice to rapidly lower the temperature. Cryotubes were immediately placed in a plastic bag and submerged in the ethanol for 2 min. Then cryotubes were stored on dry ice until placed at −80°C upon returning to the laboratory. In total, 22 samples were analyzed from 14 sites (Fig. 1). Samples were identified by their collection site number. They are designated with the suffix S if only a single sample was collected at the site. When enough mat biomass grew at a site, two samples were collected, and the upstream and downstream samples (30–50 meters apart) were designated as U and D, respectively.

Total dissolved nitrogen and phosphorus, nitrate, ammonium, depth, surface flow velocity, canopy cover, conductivity, temperature, dissolved oxygen, alkalinity, and pH were measured at each site (collection and analysis details in supplementary methods). After a mat sample was collected for DNA extraction, ~1 g of the remaining mat was collected to measure anatoxin-a using liquid-chromatography mass spectrometry (LC-MS; details in supplementary methods). During field collection and LC-MS preparation, three anatoxin-a samples were inadvertently damaged, therefore only 19 out of 22 samples were measured for anatoxin-a.

### DNA extraction and sequencing

DNA was extracted from samples using a MoBio (Carlsbad, CA, USA) DNeasy PowerBiofilm kit. Frozen mat samples were thawed at room temperature for 0.5 h, and ~0.15 g of mat removed for DNA extraction. The DNA extraction followed manufacturer’s protocol, except the cell lysis step in the protocol was modified to 5 min of bead beating and submersion for 30 min in a 65°C water bath. DNA was eluted into doubly distilled H_2_O, and sequenced on an Illumina HiSeq 4000 (San Diego, CA, USA) with 150 bp paired-end reads at the QB3 Genomics Sequencing Laboratory (http://qb3.berkeley.edu/gsl/, Berkeley, CA, USA).

### Metagenome assemblies, binning, and analyses

Reads were filtered to remove Illumina adapters and contaminants with BBtools, then trimmed with SICKLE (https://github.com/najoshi/sickle) using default parameters. Assembly and scaffolding were performed by IDBA_UD [45]. For assembled scaffolds longer than 1 kbp, protein-coding genes were predicted with Prodigal in the meta-mode [46]. Predicted genes were then annotated against KEGG [47], UniRef100 [48], and UniProt using USEARCH [49]. Genomes were binned manually using coverage, GC content, single copy genes, and taxonomic profile with ggKbase (ggkbase.berkeley.edu), as described in Raveh-Sadka et al. [50]. Genomic data (draft genomes and metagenomic reads) used for analyses have been deposited to NCBI under BioProject number PRJNA448579.

The taxonomic composition of the microbial assemblage in samples was investigated by constructing a maximum likelihood phylogenetic tree with RAxML [51] using the ribosomal protein S3 (rpS3) gene. Once the tree was built, eukaryotic chloroplast rpS3 clusters and rpS3 clusters belonging to endosymbiotic cyanobacteria of Rhopalodiaceae diatoms were excluded from further analyses. Then, average nucleotide identity (ANI), 16S ribosomal RNA, *gyrB*, and *rbcL* genes were used to investigate the diversity and taxonomy of the dominant *Microcoleus* genomes in each sample (details in supplementary methods). In genomes >70% complete and with <10% contamination, according to CheckM (Table S5; [52]), genes for different metabolisms, phosphorus cycling, and anatoxin-a biosynthesis were predicted using the annotation process described above, as well as hidden Markov-models and read mapping (details in supplementary methods).

Beta diversity, the turnover in species between sampling sites, was calculated with the R package, vegan [53] based on the presence or absence of rpS3 clusters using the *β*_sim_ metric, which minimizes the influence of high species richness differences between samples on the beta diversity metric [54, 55]. Minimum *β*_sim_ values of zero indicate identical species lists between samples, and maximum values of one indicate no shared species between samples. All clustering of data used Ward’s method [56] in the R package vegan [53].

### Spatial, environmental, and statistical analyses

Spatial and environmental distribution patterns of the dominant *Microcoleus* in the mats and ANI between their genomes were also investigated. The pairwise distances along the river network (i.e., the paths between points are constrained to river channels) between all sampling sites were calculated using ArcGIS 10.2 [57] and compared to ANI percentages with a generalize additive model (GAM) [58]. An ordination using principal components analysis (PCA) was conducted on scaled environmental variables after they were centered on their mean and standardized by their standard deviation. Relationships between the scaled environmental dissimilarity among the sites and the ANI percentages of *Microcoleus* genomes were investigated with permutational multivariate ANOVA (PERMANOVA; [59]) and generalized dissimilarity models (GDM). The GDM method tests associations between genetic relatedness and environmental conditions with nonlinear models between genetic and environmental dissimilarity matrices [60, 61]. We started with all the environmental variables listed in Table S6, excluded variables that were correlated with one another, then used backwards selection to remove variables and assess the change in deviance explained by the model until all variables with no effect on model deviance were removed. We also looked at the ANI relationship of genomes that came from upstream and downstream samples in the same site with a Mann–Whitney non-parametric statistical test. These analyses were performed in R v3.4.2 [62] using the mgcv, vegan [53], and gdm [63] packages.

## Results

### Diversity of dominant mat-forming cyanobacteria

**Fig. 2:**
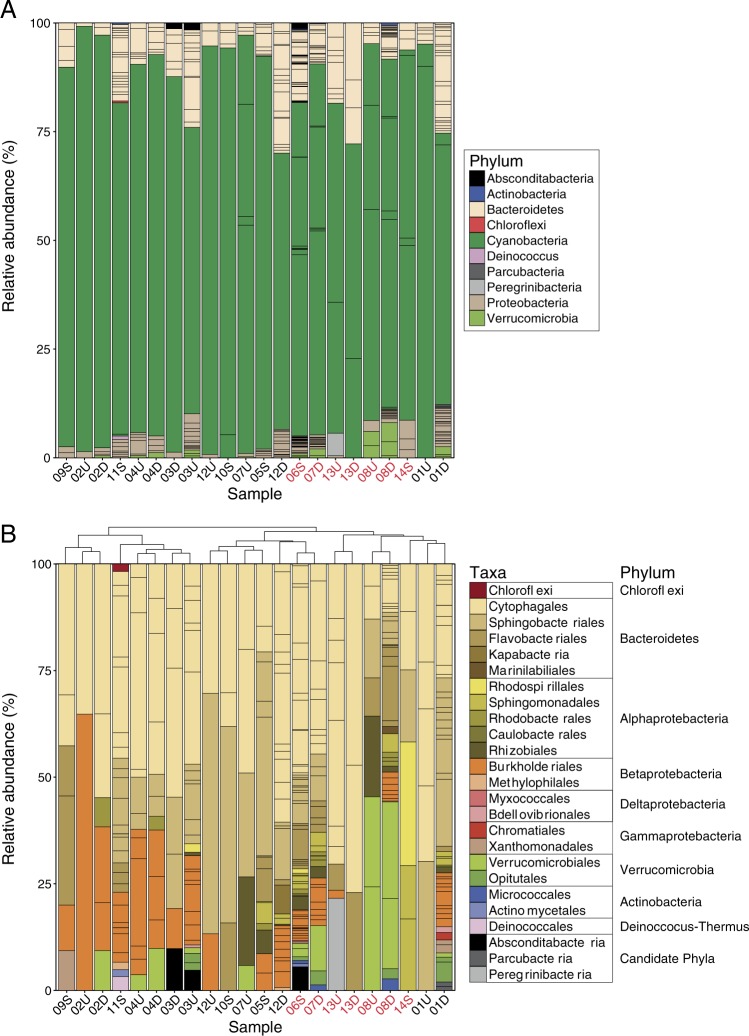
Relative abundance of ribosomal protein S3 (rpS3) sequence clusters in samples. **a** Percent relative abundance of rpS3 sequence clusters in samples colored by phyla. **b** Percent relative abundance of non-cyanobacterial rpS3 clusters in samples colored at a variety of different taxonomic levels that indicate the best classification given the bin novelty. Columns are clustered by Ward’s distance, and samples in red indicate the recovery of the anatoxin-a gene cluster in that sample

Cyanobacteria were the most abundant organisms in all mat samples, comprising 62–98% of the relative abundance of rpS3 clusters within each sample (Fig. 2a). Sixteen Cyanobacteria rpS3 clusters were identified (Figures S1 and S2). Six of these formed a clade with *Oscillatoria nigro-viridis* PCC7112, *Microcoleus vaginatus* FGP-2, and *Oscillatoria* sp. PCC506, and belong to the order Oscillatoriales (Figure S1). All six rpS3 clusters were abundant in their samples.

**Fig. 3:**
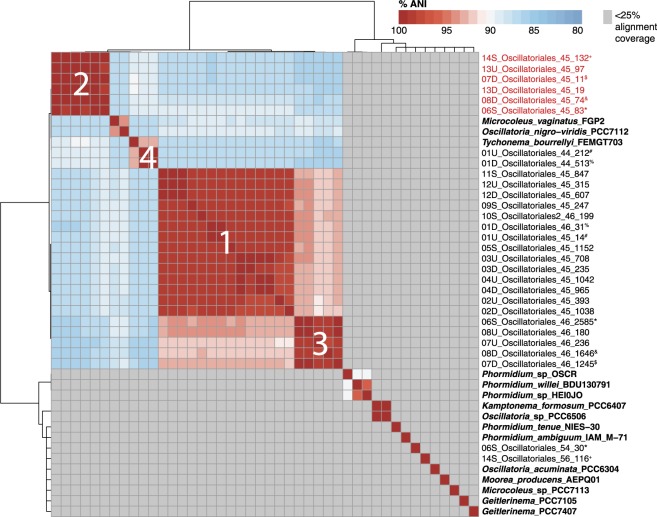
Species clusters of Oscillatoriales genomes. Heatmap shows percent average nucleotide identity (ANI) of Oscillatoriales genomes from samples and 15 reference genomes (in bold). ANI values were clustered using Ward’s distance and cluster into four *Microcoleus* species groups with <96% ANI between the clusters. Genome comparisons with alignment lengths of <25% of genome length are indicated in gray, and ANI was not calculated for these comparisons. The first number of the genome name identifies the sample location (see Fig. 1), and the latter two numbers indicate the GC content and genome coverage (the first row, therefore, designates an Oscillatoriales collected from site 14S that had 45% GC content and 132× coverage). Genome names in red contain the anatoxin-a gene cluster. Matching superscript symbols after the genome names indicate genomes that were binned from the same sample

Four Oscillatoriales species were identified based on ANI clustering of the reconstructed genomes (Fig. 3), using a species level ANI similarity threshold of >96% ANI between genomes in the same species [64]. In six samples (01U, 01D, 06S, 07D, 08D, and 14S), a second or third Oscillatoriales genome was also recovered, and the additional recovered genome belonged to a different Oscillatoriales species cluster, often occurring at lower coverage (Fig. 3).

The four Oscillatoriales species (Fig. 3) belong to the genus *Microcoleus*. Phylogenetic trees with the 16S ribosomal RNA gene (16S rRNA), gyrase subunit B (*gyrB)*, and ribulose bisphosphate carboxylase large chain *(rbcL)* genes clustered the Oscillatoriales genomes into four clades within the genus *Microcoleus* (Figure S3). The 16S rRNA tree located all sample sequences within the *Microcoleus sensu stricto* clade (Figure S3), defined in Strunecký et al. [14]. The three trees each placed the species near the *Microcoleus vaginatus* FGP2, *Oscillatoria nigro-viridis* PCC7112, and *Tychonema bourrellyi* FEMGT703 reference strains (Figure S3). Although placed near reference strains on the phylogenetic trees, the four *Microcoleus* species are distinct from all previously published reference genomes. Only three of the reference genomes (*Microcoleus vaginatus* FGP2, *Oscillatoria nigro-viridis* PCC7112, and *Tychonema bourrellyi* FEMGT703) aligned with >25% genome length to any of the Oscillatoriales genome. The highest ANI value between a sample and reference genome was between *Tychonema bourrellyi* FEMGT703 and *Microcoleus* species 4, with 93% ANI.

Two of the Oscillatoriales genomes, 06S_Oscillatoriophycideae_54_30 and 14S_Oscillatoriales_56_116, did not align over >25% to any other sample genome or reference genome, and therefore are not closely related to the four *Microcoleus* species (Fig. 3). The rpS3 genes for these samples, rpS3 232 and 114, respectively (Figures S1 and S2), cluster separately from the other relative high abundance rpS3 sequences that cluster with the *Oscillatoria nigro-viridis* PCC7112, *Microcoleus vaginatus* FGP-2, and *Oscillatoria* sp. PCC506 reference rpS3 sequences, and so these genomes likely belong to a genus other than *Microcoleus* within the order Oscillatoriales.

### Spatial distribution of *Microcoleus* species

**Fig. 4:**
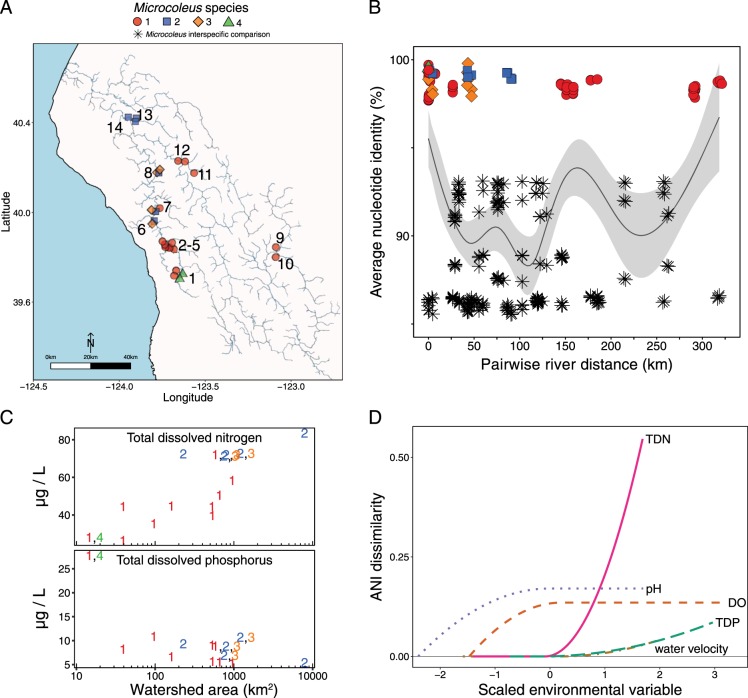
Spatial and environmental relationships of four *Microcoleus* species. **a** Location of genomes from different *Microcoleus* species, which are indicated with a different shape and color (genome locations were jittered to prevent overlap of multiple genomes recovered at a single site). Site numbers are indicated in black (see Fig. 1). **b** Pairwise comparisons of distances between collection sites along the river network and average nucleotide identity (ANI) percentage between all genomes. Comparisons between genomes that came from the same *Microcoleus* species (within species comparisons) are identified by colors and shapes, all comparisons between genomes from different *Microcoleus* species are indicated by a black star. The black line shows GAM predictions for all comparisons (within and between species) surrounded by 95% confidence intervals in light gray. **c** Dissolved nutrient concentrations and *Microcoleus* species distributions at different sampling sites. Each number on the figure indicates what *Microcoleus* species were present at a site, and colors correspond to the *Microcoleus* species in panel **a**. If more than one species were present at a site, the species numbers are separate by commas. The *x*-axis represents the upstream watershed area at each site. **d** Generalized dissimilarity model (GDM) I-spline partial effects of environmental variables on ANI. The maximum height of each I-spline curve is proportional to the amount of ANI variation associated with that variable. The shape and slope of the curve shows the changing relationship between ANI values and environmental variables along the range of values for each variable

While *Microcoleus* species 1 was distributed throughout most sites sampled in the watershed, *Microcoleus* species 2, 3, and 4 were more spatially restricted (Fig. 4a). *Microcoleus* species 1 genomes were recovered from all sites sampled in the watershed except for the northern sites 13 and 14. *Microcoleus* species 2 was found in the northern sites (13 and 14), and in the middle reaches of the South Fork Eel (6–8) and was absent in the southern or eastern sites. Species 3 was found in three sites in the South Fork Eel (6–8), and species 4 came only from site 1, a creek in the headwaters of the South Fork Eel.

ANI within a given species was independent of the river distance separating samples. The relationship between river distance and ANI was nonlinear (*p *< 0.01 from the generalized additive model (GAM); Fig. 4b), and is driven by the ratio of intraspecific and interspecific comparisons made at a given pairwise river distance. At distances of <25 km, 73% of samples (28/38) belong to the same ANI species cluster (Fig. 4b). Interspecific ANI comparisons (Fig. 4b, black stars) increase when sites are separated by 25–150 km. At distances >150 km many of the sites contained *Microcoleus* species 1, and so at these distances exists a positive correlation between distance and ANI percentages.

Within a *Microcoleus* species the ANI between genomes collected from the upstream and downstream locations <1 km apart within a site were higher than ANI between genomes collected from different sites (Mann–Whitney, *p* < 0.01; Figure S4). *Microcoleus* species 1, for example, contained a sub-cluster from sites 3 and 4 with 99–100% ANI (Fig. 3), all collected within 0.5 km in a single creek (Figs. 1 and 4b). The effect of distance on intraspecific ANI was only present at distances <1 km apart. When considering all ANI comparisons within a species, the relationship between distance and ANI within a *Microcoleus* species was not significant (Fig. 4b, colored symbols).

### Environmental distribution conditions of *Microcoleus* species

We sampled sites that drained from 17 to 7908 km^2^. Channels with larger drainage areas are wider, and generally receive more solar radiation. In our study, there were nutrient gradients down drainage networks as well. Total dissolved nitrogen (TDN) increased downstream from 2 to 40 μg/L in our August sampling (Fig. 4c), while total dissolved phosphorus (TDP) dropped sharply from ~25 μg/L in headwaters (draining 17 km^2^) to <12 μg/L in larger channels. Both trends were likely due to increasing microbial (algae and bacteria) demand for phosphorus and to contributions of nitrogen fixers in larger, more sunlit channels [65]. *Microcoleus* species 1 was broadly distributed across these gradients of light and TDN (Figure S5). *Microcoleus* species 2 occurred only where TDN exceeded 70 μg/L. Species 3 occurred at high sunlight, high TDN sites, while *Microcoleus* species 4 was found only in dark headwaters where TDN was very low (Figure S5). Other environmental conditions (pH, dissolved oxygen, canopy cover) were also measured (Figure S5 and Table S6), although some of these are themselves responses to community photosynthesis (e.g. pH and dissolved oxygen), which varies spatially with light and temporally over diel and longitudinal scales.

The generalized dissimilarity model (GDM) found the strongest association between TDN and ANI (Fig. 4d) and explained 33% of the variance in the ANI. In a GDM model, each variable’s I-splines are similar to partial regressions, and the maximum height of the I-spline represents the strength of that variable’s association with genetic distance [60, 61]. Above TDN values of 60 µg/L, *Microcoleus* species 2 and 3 are most common (Fig. 4c). This trend was driven by high TDN concentrations at sites 13 and 14, where only *Microcoleus* species 2 was recovered (Fig. 4a–c). Additional variables included in the model were dissolved oxygen, pH, TDP, and water velocity, while other environmental variables did not improve the model’s fit. The TDP effect is likely driven by site 1, which had the highest TDP value (Fig. 4c and Table S6) and was also the only location where *Microcoleus* species 4 was found. Dissolved oxygen and pH were also significant variables in the GDM model. These variables are both driven by photosynthesis, which fluctuates daily in the river, making their ecological interpretation challenging. Their inclusion in the model is likely a result of lower pH and dissolved oxygen values at sites 1 and 13 (Figure S5 and Table S6). These are both creek sites, and probably have lower photosynthesis rates than the sunnier mainstem sites.

### *Microcoleus* capacity for biosynthesizing anatoxin-a

**Fig. 5:**
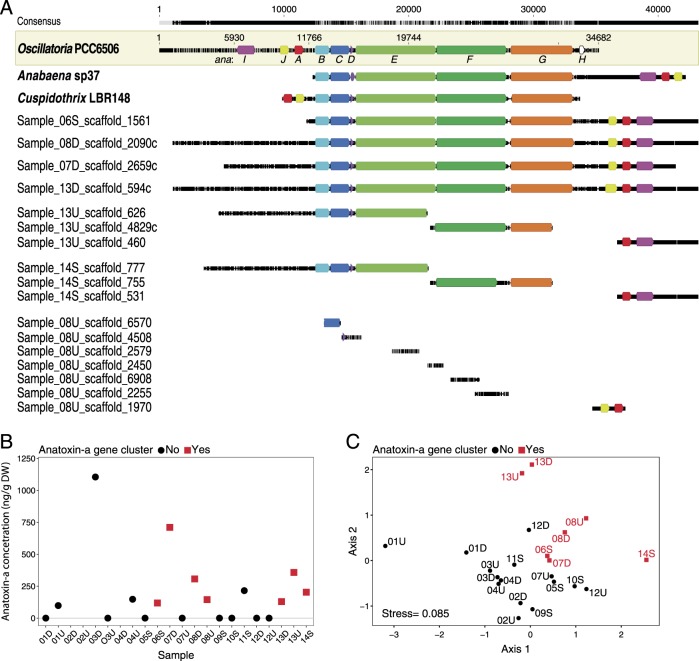
Anatoxin-a biosynthesis gene cluster and concentration in *Microcoleus* mats. **a** Anatoxin-a gene cluster from three reference sequences (bold) and samples 06S, 14S, 13U, 08D, 07D, 13D, and 08U. Sample scaffolds were mapped to *Oscillatoria* PCC6505, and gene annotations added at 50% identity to one of the 3 reference sequences. Different *ana* genes are different colors. **b** Anatoxin-a concentrations in *Microcoleus* mat samples measured by liquid chromatography and mass spectrometry and standardized by dry weight (DW). Anatoxin-a was not measured in samples 02U, 02D, and 04D due to samples being inadvertently destroyed. **c** Non-metric multidimensional scaling (NMDS) plot using Bray–Curtis dissimilarities of non-cyanobacterial assemblage showing samples with (red squares) and without (black circles) the anatoxin-a gene cluster

The anatoxin-a biosynthesis gene cluster (*anaA*–*anaJ*) was recovered from seven samples (Fig. 5a). LC-MS analyses detected anatoxin-a in 11 out of 19 samples (Fig. 5b), with median and maximum concentrations of 153 and 1104 ng anatoxin-a/g DW, respectively. The anatoxin-a gene cluster was positively associated with LC-MS detection of anatoxin-a (Fisher exact test, *p* < 0.05), although in four samples where anatoxin-a was detected, the gene cluster was not recovered (Fig. 5b).

Only one of the four *Microcoleus* species is predicted to produce anatoxin-a, as the anatoxin-a gene cluster was only identified in the six genomes in *Microcoleus* species 2 (Figs. 3 and 5a). Read mapping to the anatoxin gene cluster in sample 06S (scaffold PH2015_06S_scaffold_1561) detected the anatoxin biosynthesis genes only in samples 06S, 07D, 08U, 08D, 13U, 13D, and 14S (Table S7). In sample 08U the genes were at low coverage and on short scaffolds (~1 kbp) and could not be binned into a genome. In this sample, only the *anaC*, *J*, and *I* genes were assembled and annotated. Other scaffolds in sample 08U mapped to the PCC6506 gene cluster, but the genes were not annotated on the assembled scaffolds at >50% nucleotide identity.

All anatoxin-a gene functional annotations from these sequences matched those previously reported [35, 37, 38, 66]. Most of the *ana* genes shared 88–94% nucleotide identity to the PCC6506 reference sequence (Table S8). The nucleotide sequences of *anaC* were identical among samples, but the nucleotide sequences of other genes in the cluster were more variable among samples (e.g. *anaE*, *F*, *G*, and *J*; Table S8). The arrangement of the *anaB-G* genes was similar to the three reference gene clusters (Fig. 5a). However, *anaA*, *I*, and *J* genes were located on the opposite end of the cluster compared to PCC6506, and closer to *anaG* than the two Nostocales reference gene clusters (*Anabaena* and *Cuspidothrix*) (Fig. 5a). In samples 14S and 13U, *anaJ* was not assembled, but read mapping confirmed its presence in these samples. The *anaH* gene (annotated previously as a transposase) was not identified in any samples. The *anaG* gene was ~1670 base pairs shorter than in the three reference sequences. This missing region contained the ~300 base pair methyltransferase domain, proposed to encode the formation of either anatoxin-a or homoanatoxin-a [35, 67]. All samples also lacked the *anaK* gene, which is proposed to encode the formation of dihydroanatoxin-a [67].

### Co-occurring microbes, energy flow, and nutrient cycling in mats

Interestingly, the non-cyanobacterial microbial assemblage composition differed (*p* < 0.01) in samples that contained the anatoxin-a gene cluster (Fig. 5c). This relationship is primarily driven by fewer Betaproteobacteria (*p* < 0.01) in all samples without the anatoxin-a gene cluster (Figs. 2b and 5c). However, there were no differences in microbial assemblage composition among samples with and without LC-MS detection of anatoxin-a (*p* = 0.089). Each sample included a relatively unique set of rpS3 sequence clusters (Figure S2), resulting in low species overlap (high beta diversity) among the sites, with mean and median *β*_sim_ values of 0.73 and 0.8. Additionally, 25% of pairwise comparisons among sites had *β*_sim_ values of 1, indicating no shared rpS3 clusters among these samples.

**Fig. 6:**
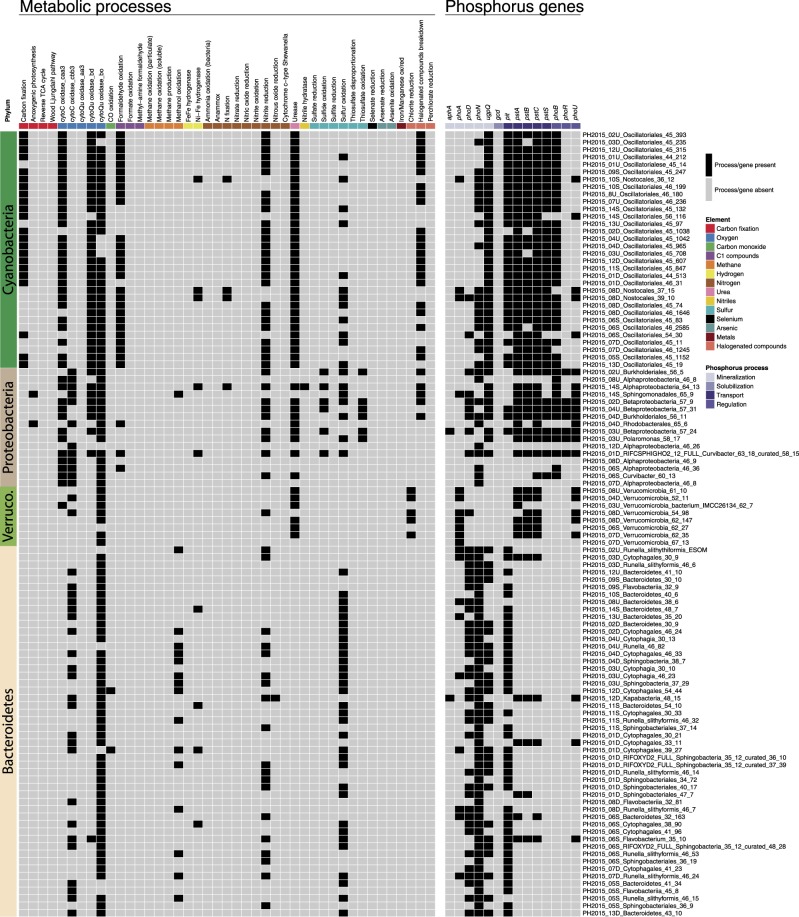
Presence or absence of metabolic processes and phosphorus acquisition and transport genes in reconstructed genomes. Each genome is a row, with the genome name listed to the right of the heatmap. Column colors indicate different metabolism types (left panel), or genes involved in phosphorus acquisition and transport (right panel). A process or gene that is predicted in a genome is indicated by a black square. The phyla of the genomes is listed to the left of the heatmap (Verruco. = Verrucomicrobia)

The most abundant non-cyanobacterial taxa in the *Microcoleus*-dominated mats were Bacteroidetes (97 rpS3 clusters), occurring in all 22 samples and representing 35–100% of the non-cyanobacterial assemblage (Fig. 2 and S2). Organisms in the phylum Bacteroidetes had genes for few metabolic processes, with most genomes containing genes for carbon and sulfur oxidation (Fig. 6).

Along with Bacteroidetes, Proteobacteria (77 rpS3 clusters), primarily Burkholderiales and Sphingobacteriales, were also common in samples (Fig. 2). Organisms in Proteobacteria had the most diverse sulfur metabolic potential in the mats. Genes for sulfide oxidation (flavocytochrome c sulfide dehydrogenase), sulfur oxidation (sulfur dioxygenase), and thiosulfate oxidation (*soxB*, *C*, and *Y*) were identified in genomes from both Alpha and Betaproteobacteria (Table S9).

Verrucomicrobia (8 rpS3 clusters) were detected in 10 samples at relative abundances sometimes exceeding >40% of the non-cyanobacterial assemblage (Fig. 2, S1, and S2). These organisms contained genes predicted to encode for carbon oxidation and nitrite reduction and are the only organisms found in the mats with genes encoding chlorite reduction (Fig. 6).

Minor members of the community included bacteria from Actinobacteria, Deinococcus-Thermus, Chloroflexi, *Canditatus* Kapabacteria, and three Candidate Phyla Radiation bacterial groups (CPR; Figure S1). The four CPR rpS3 clusters that occurred at low abundance were identified as Peregrinibacteria (PER), Absconditabacteria (SR1), and Parcubacteria (OD1) (Figure S1).

Overall, the most abundant metabolisms in assembled genomes were phototrophic, heterotrophic, nitrogen, and sulfur metabolisms (Fig. 6 and Table S9). Although genes associated with nitrogen fixation, hydrogen oxidation, halogenated compound breakdown, and formaldehyde oxidation were also detected, the most common processes were carbon fixation, carbon oxidation, nitrite reduction, utilization of urea, and sulfur oxidation (Table S9). In addition to carbon fixation, Oscillatoriales genomes contained genes for formaldehyde oxidation, urea breakdown, nitrite reduction, sulfur oxidation, and halogenated compound breakdown. Across the four *Microcoleus* species, there was little variation in metabolic potential (Fig. 6).

Phosphorus can be limiting in river ecosystems and concentrations were low at most sites (<10 µg/L; Table S6). Diverse phosphorus uptake and transport genes were annotated in the reconstructed genomes (Fig. 6 and Table S10). The non-cyanobacterial assemblage contains genes for alkaline phosphatase, while the most common phosphorus mineralization genes for *Microcoleus* were acid phosphatase, *phoN*, and glycerophosphodiesterase, *ugpQ*. The low-affinity inorganic phosphorus transport gene, *pit*, was most frequently identified in Cyanobacteria and Bacteroidetes genomes, while all Verrucomicrobia and most Proteobacteria lacked this gene. *Microcoleus* also contained the high affinity phosphate transport, *pst*, genes, which are up-regulated at low inorganic phosphorus concentrations [68]. Unlike other phyla, Verrucomicrobia only possessed *phoA* and the *pst* transporter genes. The phosphorus-solubilization gene, *gcd*, was absent from all genomes.

## Discussion

This study is the first to our knowledge to perform genome resolved metagenomics on potentially toxigenic benthic cyanobacterial mats. By sampling mats once at sites distributed throughout an entire watershed, we took a complementary approach to the other published study on *Microcoleus* microbial communities by Brasell et al. [29], who sampled the same site longitudinally over time. We recovered genomes of four *Microcoleus* species, which begins to describe and map the diversity of *Microcoleus* species in the Eel River watershed.

### *Microcoleus* taxonomy

Revisions to the *Phormidium* and *Microcoleus* genera over the last decade challenge taxonomic assignations of potentially toxigenic benthic cyanobacteria. Our results assign morphologically distinct, recognizable Oscillatoriales mats, which are common in the Eel River and had formerly been designated as *Phormidium*, to the genus *Microcoleus*. By comparing our phylogenies of the rpS3, 16S rRNA, *gyrB*, and *rbcL* genes, we can place our dominant *Microcoleus* genomes in a clade with *Microcoleus* reference genomes *Microcoleus* FGP2 and *Oscillatoria nigro-viridis* PCC7112 on the phylum-wide cyanobacterial tree constructed by Calteau et al. [38]. (Molecular phylogenies show the PCC7112 genome, though still named *Oscillatoria*, belongs in the *Microcoleus* genus [15, 16, 69]).

Calteau et al. [38] did not include many of the reference sequences used by Strunecký et al. [14] to re-define the *Phormidium* and *Microcoleus* genera. Therefore, the Calteau et al. tree cannot place our organisms in a genus. Comparing 16S rRNA sequences from our genomes to sequences used in Strunecký et al. [14] confirmed the placement of our Oscillatoriales genomes in *Microcoleus*. We did not expect *Microcoleus* species 4 to be most similar to the *Tychonema bourrellyi* FEMGT703 genome (Fig. 3 and S3), because bacteria of the genus *Tychonema* are planktonic and found in lakes. The close phylogenetic relationship between *Tychonema* and *Microcoleus* [70, 71] suggests that *Tychonema* may not be constrained to planktonic habitats and supports Komárek et al.’s [15] call for taxonomic revision of *Tychonema*. Based on our genomic results, we conclude the dominant Oscillatoriales genomes assembled from our mats are within the genus *Microcoleus*, rather than in *Phormidium* or *Tychonema*.

### Spatial distribution and possible environmental controls of *Microcoleus* species

Different between-species versus within-species patterns emerged from the spatial distribution of *Microcoleus* genomes in our samples (Fig. 4b and S4). *Microcoleus* species 1 was broadly distributed across the watershed, while species 2 and 3 were found only in the northwestern (most downstream) sites, where species 1 was often absent (Fig. 4a). ANI similarity decreased sharply from 0–25 km river network distances between sites due to the increasing number of between species comparisons in sites separated by greater distances (Fig. 4b). Since we found no linear relationship between ANI for species comparisons and distance at the watershed scale, it appears that geographic isolation is not the main driver of species distributions in the watershed.

Spatial distances appear to affect ANI within a *Microcoleus* species. *Microcoleus* strains from sites separated by <1 km have higher ANI that those from sites that were separated by >1 km (Figure S4). The lower ANI for strains from more distant sites may be explained by the riffle and pool morphology of the Eel River. *Microcoleus* primarily grow in faster-flowing riffle habitats, with the next downstream riffle separated by a slower-flowing pool habitat. This riffle-pool pattern repeats downstream to the ocean. Since riffles are separated by pools seemingly inhospitable for *Microcoleus*, the riffle-pool morphology may be a barrier to gene flow, isolating cells in one riffle from other riffles downstream. Countering this, dispersal by downstream flow could bring closely related strains into close physical proximity, enabling homologous recombination that counters strain divergence. Alternatively, region-specific environmental parameters could shape the spatial distribution of strains within species across the watershed.

Environmental factors that could affect benthic cyanobacteria change systematically down all river drainages. In the Eel watershed, there are also east-west gradients associated with variation in climate (maritime influences wane as one moves east) and lithology (the western tributary, the South Fork Eel, flows over deep weathered shales and sandstones, which stabilize summer flow relative to flows in mainstem or eastern tributaries that drain less permeable, clay-rich mélange substrate [72, 73]). Detecting limiting environmental factors and thresholds associated with the proliferation of toxin-producing *Microcoleus* is challenging [31], as different taxa may proliferate in different conditions, yet be indistinguishable from one another macroscopically and even microscopically. In Spain, multiple *Phormidium*-like species were defined using 16S rRNA genes (also clustering with *Oscillatoria nigro-viridis* PCC7112, as do our samples), although they had been lumped together as a single group using morphological characteristics determined by light microscopy [74]. The three Spanish species inhabited different sites along nitrogen and phosphorus gradients. In the Eel River, we also found strong correlations of nutrient concentrations with the distribution of the different *Microcoleus* species. There was, however, little variation in metabolic or phosphorus genes within the different *Microcoleus* genomes (Fig. 6). How these organisms may respond to different nutrient environments may be driven by differences in enzyme kinetics, gene regulation, acquisition and transport genes, interactions with other micro organisms, or other factors not evident from their genomes alone.

One important difference among the *Microcoleus* genomes was that the gene cluster predicting anatoxin-a biosynthesis was found only in *Microcoleus* species 2. For reasons that remain unknown, this species was found in sites with higher TDN concentrations. Cyanobacterial blooms have been long associated with increased nutrient concentrations, but only recently have we learned more about how nutrients influence cyanotoxin production (e.g. [41, 75]). Evidence is mounting that *Microcystis* spp. blooms in Lake Erie switch to non-microcystin producing genotypes later in summer as nitrogen concentrations decrease [41, 76]. Given the different molecular composition of cyanotoxins, differential responses of cyanotoxin production to nutrient availability likely depend on the stoichiometry of different cyanotoxin molecules [75]. For example, microcystin molecules contain ten nitrogen atoms while anatoxin-a molecules contain only one, so nitrogen may be less important for anatoxin-a than for microcystin biosynthesis. Although riverine Oscillatoriales in New Zealand, Spain, and California are spatially correlated with nutrients, links between nutrients, toxic genotypes, and anatoxin-a production remain unclear.

### Anatoxin-a and the microbial assemblage

Cyanobacterial blooms change environmental conditions, in part through compounds they produce, which then shape microbial community composition. In lakes, cyanobacterial blooms alter assemblages of other microbes [77, 78], with impacts that depend on the species of cyanobacteria dominating the bloom [79, 80]. River environments differ from lakes, for example, in ratios of sediment surfaces to water volume, or by virtue of unidirectional, often turbulent river flows. Nevertheless, non-cyanobacterial microbes associated with cyanobacteria in the Eel River (this study) and in New Zealand rivers [29] resembled microbial consortia members, reported in lake plankton (e.g. [78, 79, 81]), at least at the phylum level. For example, all have high abundances of Proteobacteria and Bacteroidetes, although the physiology of the specific species present may differ substantially within a phyla. Further research is needed to determine the extent of similarity between consortia involved in lake planktonic blooms and riverine benthic cyanobacterial mats.

The non-cyanobacterial assemblage associated with *Microcoleus* species 2, which possessed the anatoxin-a biosynthesis gene cluster (Figs. 2b and 5c), was different from assemblages co-occurring with the three other *Microcoleus* species. While it is possible that certain non-cyanobacterial organisms select *Microcoleus* species with genes for toxin production, we think it is more likely that the dominant mat-forming *Microcoleus* species select for different co-occurring microbes. For example, there could be selection within consortia for bacteria with the capacity to degrade cyanotoxins. Microcystin concentrations have been shown to affect bacterial assemblage composition [82]. However, the relevance of this finding to the current study is uncertain because microcystin is a larger molecule with more nitrogen than anatoxin-a. Thus, other *Microcoleus*-derived molecules with higher energy or nutrient content probably have a greater impact on overall community composition than anatoxin-a.

The presence of anatoxin-a molecules was confirmed by LC-MS in all samples with the anatoxin-a biosynthesis gene cluster. In four samples, however, we detected anatoxin-a with LC-MS but did not recover the anatoxin-a gene cluster (Fig. 5b). Within-mat spatial heterogeneity and low abundances of toxic organisms may have prevented our detection. Production of anatoxin-a can vary over centimeter and hourly scales and is often driven by changes in the abundance of toxic genotypes [40]. Because we were not able to sample the same location on the cobble for both the DNA and LC-MS samples, it is possible that in these four cases, our DNA samples did not contain many toxin-producing genotypes, while the LC-MS sample did. The relationship between anatoxin-a biosynthesis gene copies and anatoxin-a concentration is variable (*r*^2^ of ~0.25 [40]). Therefore, we do not expect strong correlations between the metagenomic recovery of anatoxin-a biosynthesis genes and LC-MS concentrations. Finally, metagenomic methods may not have assembled the anatoxin-a gene cluster. This could occur if toxin-producing genotypes were present at low abundances in the DNA samples or due to genome incompleteness, for example, if a gene cluster included a repeat region, which did not assemble.

### Transformations of carbon, nitrogen, and phosphorus in mats

Overall, the mats studied here are metabolically simple, with few anaerobic or non-carbon metabolisms. We expected to find more anaerobic metabolic genes in the assemblages, as other cyanobacterial mats have fluctuating oxygen levels and [30] harbor organisms with the potential for anaerobic metabolism [29]. For example, a prior study of river-associated *Microcoleus* mats showed diel fluctuations of oxygen concentrations [30] that could, under some circumstances, lead to permanent anoxic regions similar to those that occur within cyanobacterial mats on fine sediments [83, 84]. The low abundance of organisms with anaerobic metabolisms in the Eel River system may be explained by the transient nature of anaerobic regions in thin mats growing in the relatively fast flowing riffles where turbulent flow rapidly resupplies oxygen. The microbial diversity and photosynthetic and carbon oxidation metabolisms in Eel River *Microcoleus* mats were similar to those in planktonic cyanobacterial blooms [79, 82, 85], suggesting that environmental factors that shape consortia in planktonic and benthic cyanobacterial blooms may be similar when benthic cyanobacteria grow attached to cobble surfaces in fast flowing water.

The abundant organisms in *Microcoleus* mats clearly derive their energy from photosynthesis and carbon oxidation in an aerobic environment. The draft genomes were, on average, 87% complete and not <70% complete (Table S5), which should suffice to identify most metabolic genes of an organism. As expected, *Microcoleus* mats were generally thicker in warmer, sunnier sites, so rates of growth and biomass accrual appear to increase with light and temperature. Photosynthetic carbon is exported from the *Microcoleus* cells to form thick extracellular polymeric substances (EPS) that give structural integrity to the mat [86]. The non-cyanobacterial assemblage in the mats, especially Bacteroidetes and Burkholderiales (some of the most common non-cyanobacterial taxa in our mats), are likely fueling their growth by metabolizing EPS-associated carbon compounds [87]. Candidate Phyla Radiation bacteria may also contribute to carbon cycling, as they have been reported in planktonic and benthic cyanobacterial blooms [88, 89].

Even though nitrogen is considered a limiting nutrient in spring and early summer in the Eel River [65, 90], few nitrogen fixing genes were annotated in bacterial genomes from our samples. This was not expected, because other epilithic periphyton assemblages in the Eel River are dominated by nitrogen fixing taxa [91], such as free-living cyanobacteria like *Nostoc* spp. and *Anabaena* spp., or endosymbiotic nitrogen-fixing cyanobacteria in diatoms in the family Rhopalodiaceae [92]. Our results echo findings from New Zealand, where Heath et al. [93] also found no nitrogen fixing genes in *Microcoleus* mats. Rhopalodiaceaen diatoms that harbor endosymbiotic cyanobacteria which fix nitrogen are often present, if not abundant, in Eel River *Microcoleus* mats, and may be a source of fixed nitrogen to *Microcoleus* cells. Our results suggest that *Microcoleus*, and most other bacteria, in the Eel River mats likely derive their nitrogen from a combination of water column sources, as well as endosymbionts of Rhopalodiaceaen diatoms (which were not considered in the current study) and the few nitrogen fixing bacteria living in the mats.

Phosphorus concentrations in both the Eel River and New Zealand streams that contain *Microcoleus* mats are often very low [7, 31]. Therefore, the acquisition of phosphorus by organisms in the mats is thought to be a limiting process for mat growth. *Microcoleus* proliferations may be explained by the many phosphorus acquisition and transporter genes detected in the genomes recovered from our mats. Extracellular phosphatase genes and *pst* transporter genes might enable *Microcoleus* strains to outcompete other organisms for phosphorus and dominate periphyton assemblages at low phosphorus concentrations. Some Proteobacteria possess the transporter *pst* genes and many Bacteroidetes are capable of producing extracellular phosphatase genes, so the whole microbial assemblage in the mats may interact both mutualistically and competitively in transforming phosphorus into bioavailable forms, which are cycled internally within the mats.

Although microbes possess phosphorus scavenging and transporting genes, they still need phosphorus to be delivered to the mat. Phosphate sorbed onto trapped sediments in mats has been proposed as an important phosphorus source for species in New Zealand rivers [30] and a similar process may occur in the Eel River mats. We did not detect the solubilization gene, *gcd*, which encodes the production of extracellular organic acids to solubilize sorbed phosphorus. Therefore, microbes may depend on daily fluctuations in pH and O_2_ levels to solubilize sorbed phosphate [30]. Alternatively, microbes may be using H^+^ excreted by various molecular pathways to locally affect pH concentrations around the cells and de-sorb phosphorus [94]. The suite of phosphorus genes possessed by organisms in the mat, entrapped phosphorus-bearing sediments by mats, and active internal recycling [27] may all prevent phosphorus limitation for *Microcoleus* cells.

## Conclusion

As humans deplete, pollute, and warm freshwater environments, cyanobacterial blooms will increase [95–97]. We need to understand how cyanobacteria affect and respond to abiotic and biotic components of their environments to predict or mitigate cyanobacterial blooms in the future. Higher nitrogen concentrations external to the mat and a unique microbial assemblage within the mat were associated with *Microcoleus* species 2, the species with the anatoxin-a biosynthesis gene cluster. We know little about whether it is the external environment or within-mat environment that determines when cyanobacterial mats will proliferate, which strains dominate, and how their metabolism and metabolites affect other organisms, including humans. Answering whether the biotic and abiotic conditions interact additively or synergistically and the strength of each control on *Microcoleus* growth and toxin production is increasingly possible with advancing molecular methods and will shed considerable light on the ecology of these organisms, so consequential for ecosystem and human health.

## Supplementary information

Figure S1

Figure S2

Figure S3

Figure S4

Figure S5

Supplementary methods

Supplementary tables
