# Supplementary material for: Impacts of microbial assemblage and environmental conditions on the distribution of anatoxin-a producing cyanobacteria within a river network
Source: ISME J. 2019 Feb 26;13(6):1618–34. doi: 10.1038/s41396-019-0374-3 (PMC6776057; doi:10.1038/s41396-019-0374-3)
Supplement: Supplementary file 2 — Figure S2 [file 41396_2019_374_MOESM2_ESM.pdf]

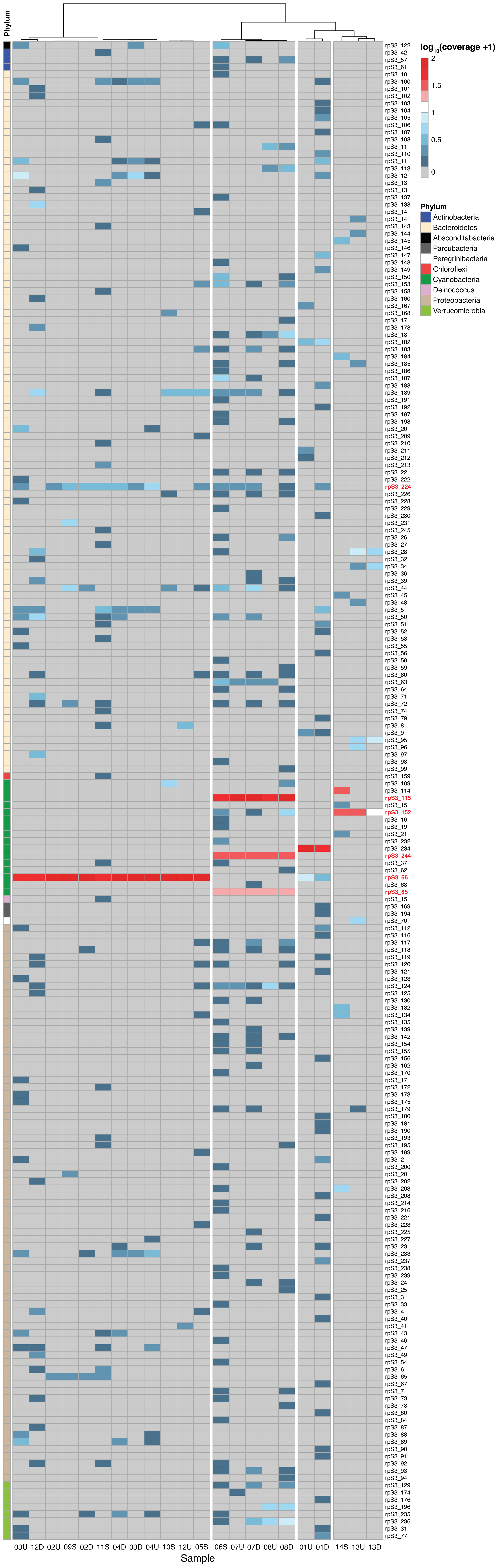

Figure S2 Coverage of ribosomal protein S3 (rpS3) sequence clusters coverage among the different samples. Each row is a unique rpS3 cluster and each column is a sample. The numbers of six rpS3 clusters mentioned in the text are given in red text. Columns are clustered by Ward's distance.
