# Supplementary material for: Impacts of microbial assemblage and environmental conditions on the distribution of anatoxin-a producing cyanobacteria within a river network
Source: ISME J. 2019 Feb 26;13(6):1618–34. doi: 10.1038/s41396-019-0374-3 (PMC6776057; doi:10.1038/s41396-019-0374-3)
Supplement: Supplementary file 3 — Figure S3 [file 41396_2019_374_MOESM3_ESM.pdf]

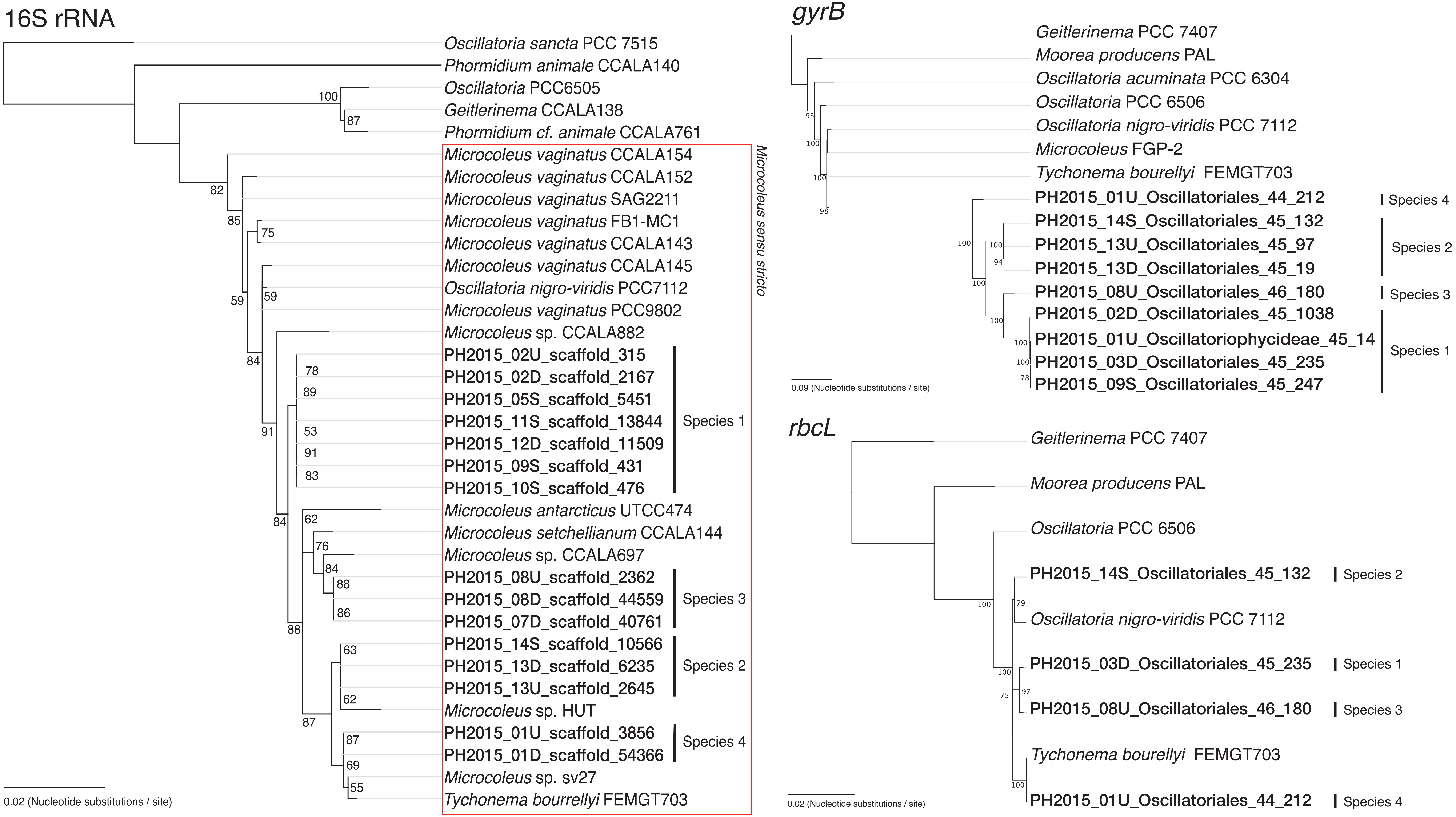

Figure S3 Maximum likelihood trees of nucleotide sequences from the 16S rRNA, *gyrB*, and *rbcL* genes. Sample sequences are in bold, and accession numbers for reference strains can be found in Table S2. The species identity of sample strains (determined by average nucleotide identity, see Figure 3 in text) is indicated on the right side of each tree. Each tree was bootstrapped 100 times and, bootstrap support is shown for nodes with support >50,. The *Microcoleus sensu stricto* clade is shown in red on the 16S rRNA tree, as was defined by Strunecký et al. (2013).
