# Supplementary material for: Impacts of microbial assemblage and environmental conditions on the distribution of anatoxin-a producing cyanobacteria within a river network
Source: ISME J. 2019 Feb 26;13(6):1618–34. doi: 10.1038/s41396-019-0374-3 (PMC6776057; doi:10.1038/s41396-019-0374-3)
Supplement: Supplementary file 4 — Figure S4 [file 41396_2019_374_MOESM4_ESM.pdf]

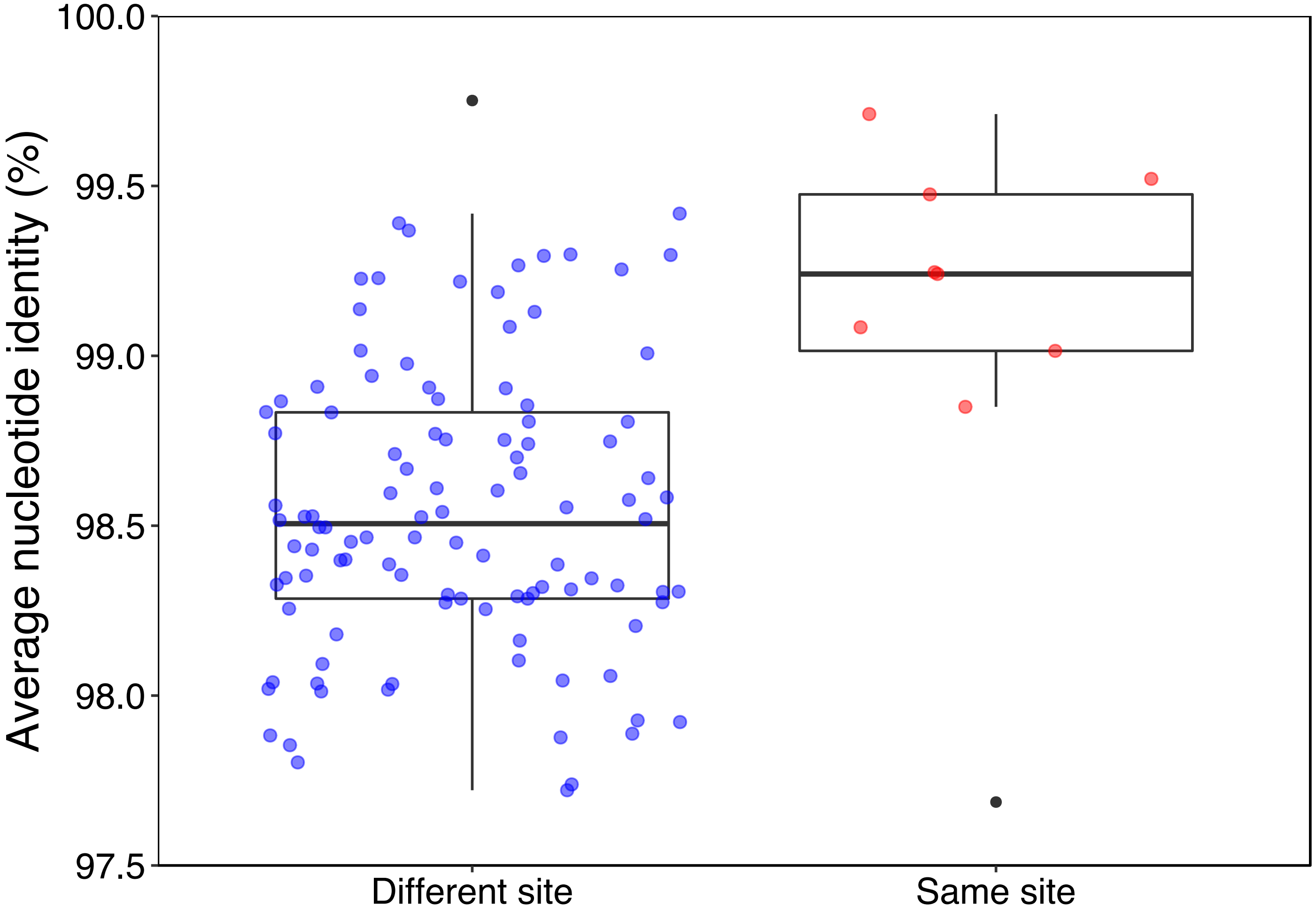


**Figure S4** Average nucleotide identity (ANI) comparisons between genomes from the same site (n= 9) and different sites (n= 108) in the Eel River watershed. Boxplots show the median and interquartile range. Whiskers extend 1.5 times the interquartile range, and outlier points are indicated in black. The mean ANI in the same site comparisons was higher than different site comparisons (Mann-Whitney test, $p < 0.01$).
