# Supplementary material for: Impacts of microbial assemblage and environmental conditions on the distribution of anatoxin-a producing cyanobacteria within a river network
Source: ISME J. 2019 Feb 26;13(6):1618–34. doi: 10.1038/s41396-019-0374-3 (PMC6776057; doi:10.1038/s41396-019-0374-3)
Supplement: Supplementary file 5 — Figure S5 [file 41396_2019_374_MOESM5_ESM.pdf]

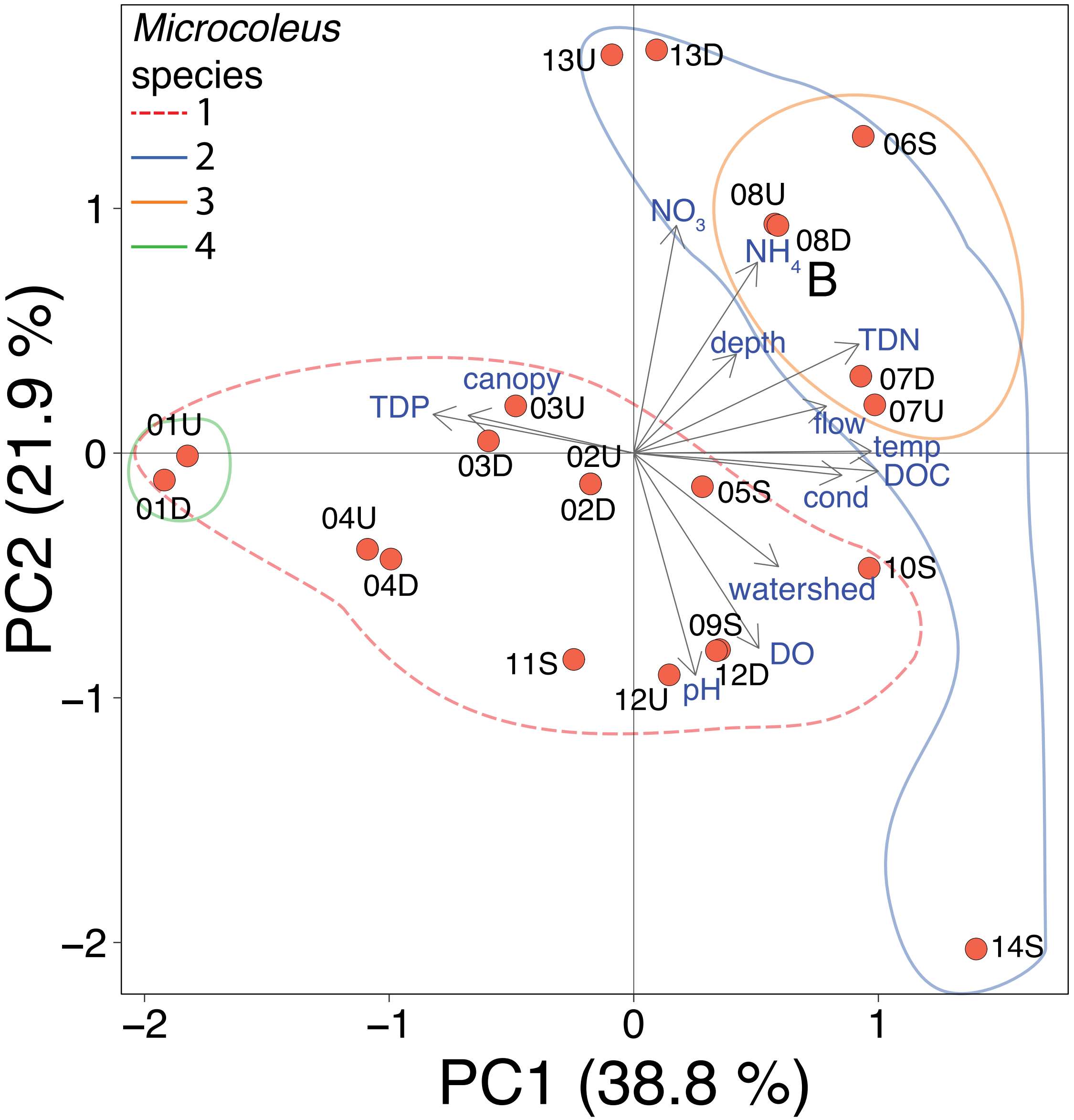

Figure S5 Principal components biplot of environmental conditions at each sampling site. Sites are labeled with red points, and environmental variables labeled with vectors. The percentage of variation described by each axis is written in parentheses. Sites where *Microcoleus* species 1-4 were found are enclosed by different colored lines for each *Microcoleus* species. Some samples contained genomes from multiple ANI clusters, and samples 02D and 02U share the same point.
