# Supplementary material for: Impacts of microbial assemblage and environmental conditions on the distribution of anatoxin-a producing cyanobacteria within a river network
Source: ISME J. 2019 Feb 26;13(6):1618–34. doi: 10.1038/s41396-019-0374-3 (PMC6776057; doi:10.1038/s41396-019-0374-3)
Supplement: Supplementary file 1 — Figure S1 [file 41396_2019_374_MOESM1_ESM.pdf]

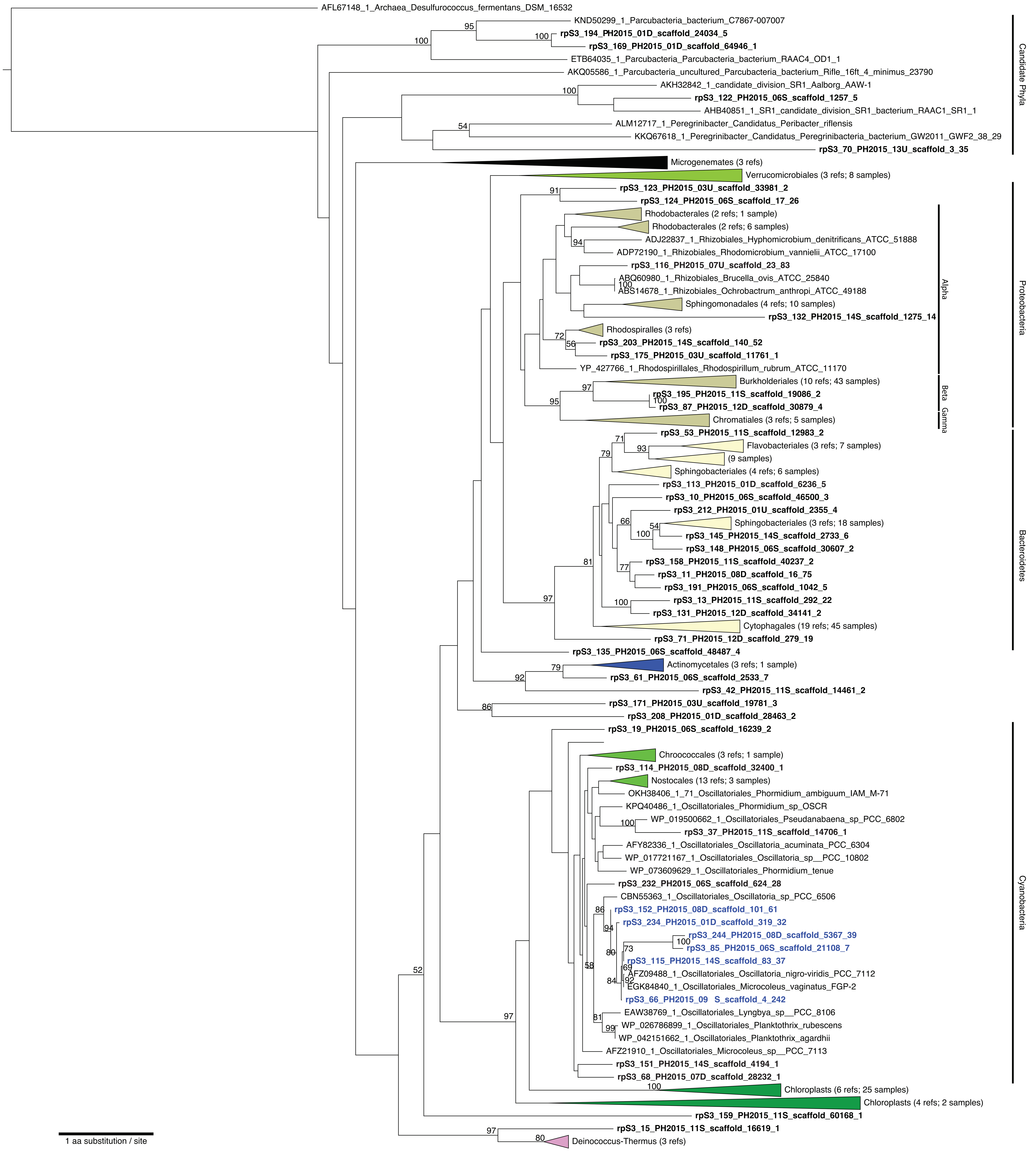


**Figure S1** Maximum likelihood (RAxML) phylogenetic tree of ribosomal protein S3 (rpS3) sequences with archaea as the outgroup. Collapsed nodes are represented with colored triangles and the number of reference and sample sequences in the collapsed node indicated in parentheses. Sample sequences are bolded and rpS3 sequences forming a clade with *Oscillatoria nigro-viridis* PCC7112, *Microcoleus vaginatus* FGP-2, and *Oscillatoria* sp. PCC506 are colored blue. Nodes with bootstrap values >50% are indicated on the tree.
