# Supplementary material for: Impacts of microbial assemblage and environmental conditions on the distribution of anatoxin-a producing cyanobacteria within a river network
Source: ISME J. 2019 Feb 26;13(6):1618–34. doi: 10.1038/s41396-019-0374-3 (PMC6776057; doi:10.1038/s41396-019-0374-3)
Supplement: Supplementary file 6 — Supplementary methods [file 41396_2019_374_MOESM6_ESM.pdf]

Supplementary Methods

Impacts of microbial assemblage and environmental conditions on the distribution of anatoxin-a producing cyanobacteria within a river network

Keith Bouma-Gregson[1,2], Matthew R. Olm[3], Alexander J. Probst[2^], Karthik Anantharaman[2*], Mary E. Power[1], Jillian F. Banfield[2,4,5,6]

[1]Department of Integrative Biology, University of California, Berkeley, CA, USA
[2]Department of Earth and Planetary Science, University of California, Berkeley, CA, USA
[3]Department of Plant and Microbial Biology, University of California, Berkeley, CA, USA
[4]Department of Environmental Science, Policy, and Management, University of California, Berkeley, CA, USA
[5]Earth Sciences Division, Lawrence Berkeley National Laboratory, Berkeley, CA, USA
[6]Chan Zuckerberg Biohub, San Francisco, CA, USA

[^]Current Address: Group for Aquatic Microbial Ecology, Biofilm Center, Department for Chemistry, University of Duisburg-Essen, Essen, Germany

*Current Address: Department of Bacteriology, University of Wisconsin, Madison, WI, USA

Corresponding author:
Jillian F. Banfield,
Energy Biosciences Building
2151 Berkeley Way
Berkeley, CA 94720-5230
510-643-2155
jbanfield@berkeley.edu

Environmental parameters at sampling sites

To measure the environmental conditions at each site, filtered water samples (0.7 µm) were collected and measured for total dissolved nitrogen (Shimadzu TOC-VCPH TC/TN analyzer), total dissolved phosphorus using persulfate acid digestion and molybdate colorimetry analysis, nitrate (Lachat QuikChem 8000 Flow Injection Analyzer), and ammonium (OPA method; [1]). At each site, we also measured depth, surface flow velocity, canopy cover (with a spherical densiometer), conductivity, temperature, dissolved oxygen (ProPlus, YSI Inc., Yellow Springs,

OH USA), alkalinity (Alkalinity Test Kit AL-DT, Hach Company, Loveland, CO USA) and pH (HI991001, Hanna Inst., Woonsocket, RI USA). The watershed area upstream of each sampling site was calculated using ArcGIS 10.2 (Esri, Redlands, CA, USA).

Microbial assemblage diversity

The taxonomic composition of the microbial assemblage in the samples was investigated using the ribosomal protein S3 (rpS3) gene. The amino acid sequences of all assembled scaffolds >1 kb were searched for the rpS3 gene using custom Hidden Markov Models (HMMs) (https://github.com/AJProbst/rpS3_trckr). The rpS3 amino acid sequences were then clustered at 99% sequence identity to approximate species-level clusters and create unique rpS3 clusters for each organism bin. The longest scaffold from each rpS3 cluster was identified, and reads from each sample were mapped onto that set using Bowtie 2 [2] allowing ≤3 mismatches per read. An organism was considered present in a sample if the rpS3 sequence was found on an assembled scaffold, or if reads from a sample mapped to the rpS3 sequence with a breadth >95%. The coverage values from read mapping for all rpS3 clusters in a sample were then normalized by the number of sequenced gigabase pairs (gbp) that went into each assembly. The relative abundance of each rpS3 gene was calculated by dividing the coverage of each rpS3 sequence in a sample, by the sum of the coverage values of all the rpS3 genes in a given sample, then multiplying by 100. Preliminary taxonomic identifications for each rpS3 cluster were derived by searching [3] the amino acid sequence against a combined database from previous publications [4, 5] and selecting the best match. Refinements to the taxonomic annotation were made using the maximum likelihood phylogenetic tree described below.

A phylogenetic tree was built to investigate the taxonomic diversity of rpS3 sequences. Reference rpS3 amino acid sequences were downloaded from NCBI and aligned with sample

sequences using MUSCLE [6]. Amino acid sites with >95% gaps after the alignment were stripped from the analysis in Geneious v8.1.8 [7], and duplicate sequences were removed. A maximum likelihood phylogenetic tree was constructed from the remaining 363 reference and sample sequences using RAxML [8] with the PROTGAMMALG amino acid evolution model and the number of bootstraps automatically determined (autoMRE).

Average nucleotide identity (ANI) was used to compare the diversity of the Oscillatoriales genomes in the mat. The quality of the 35 assembled genome bins in the order Oscillatoriales was assessed using CheckM [9]. Genomes <75% complete or with >10% contamination were excluded from further analysis. ANI was calculated on the remaining 29 genomes, and 15 reference genomes downloaded from NCBI (Table S1), with the ANIm method [10] implemented using the Python module PYANI (https://github.com/widdowquinn/pyani) [11]. This program first performs pairwise alignments of all input genomes. For each alignment, the nucleotide similarity is calculated. It then averages the nucleotide identity for all aligned regions in each pairwise comparison. As genomes differ in similarity it becomes harder for the algorithm to align the genomes and the alignment coverage decreases [12]. If less than 25% of the genome aligned, we considered the ANI results non-representative and did not include the ANI percentage in the results, and indicative of low genome wide sequence similarity. We chose 25% alignment coverage as a threshold because there was a large break in the distribution of alignment coverage values at 25%. Genomes with ANI less than 96% were considered different species [10, 12, 13].

To further investigate the taxonomy of Oscillatoriales genomes, maximum likelihood trees were constructed with nucleotide sequences from the 16S ribosomal RNA gene (16S rRNA), *gyrB* gene (gyrase subunit B), and *rbcL* gene (ribulose 1,5-bisphosphate carboxylase large subunit).

Reference strains were selected from Strunecký et al. [14] and Sciuto et al. [15], two papers that contributed to revisions of the genera *Microcoleus* and *Phormidium*. Genes were identified in the Oscillatoriales genomes from the annotation methods described above and by blasting genomes with sequences from *Oscillatoria nigro-viridis* PCC 7112. Reference and sample nucleotide sequences (Table S2) were aligned with MUSCLE [6] and non-informative sites stripped with BMGE [16]. Trees were built with RAxML [8] using the CIPRES Science Gateway [17] with the GTRCAT substitution model and 100 bootstraps for each tree.

Metabolic potential and phosphorus acquisition

Profiling of metabolic potential was performed on genome bins that passed quality filtering (>70% complete and <10% contamination according to CheckM). This resulted in 106 genomes with mean and median contamination of 1.4% and 0.6%, respectively, and mean and median completeness of 87.3% and 88.0%, respectively (Table S5). The amino acid sequences of predicted genes from genome bins were compared to TIGRFAM HMMs [18] and custom HMMs for metabolic pathways involving arsenic, C1 compounds, carbon, carbon monoxide, halogenated compounds, hydrogen, nitriles, nitrogen, oxygen, sulfur, and urea from Anantharaman et al. [4]. Cut off values for HMM scores were derived from Anantharaman et al. [4] (Table S3).

Phosphorus acquisition and transport were investigated by searching genomes for genes involved in phosphorus transport, solubilization, mineralization, and regulation using Pfam or TIGRfam HMMs (Table S4) [19]. Cutoff values were derived by downloading 21 annotated isolate genomes and searching them using the HMMs. Search results were verified with blastp against the NCBI RefSeq database (June 2017) by looking for enzyme name keywords from Table S4 indicating gene functions, and then establishing cutoff values.

Anatoxin-a gene cluster

Some, but not all, genes in the anatoxin-a gene cluster were correctly annotated via the procedures described above. Additional anatoxin-a biosynthesis genes were identified by investigating genes surrounding correctly annotated genes. To search for additional scaffolds with the anatoxin-a gene cluster, HMMs were built using hmmbuild (hmmer.org) for each gene in the anatoxin-a gene cluster using reference sequences from NCBI. All genes in the vicinity of the above identified genes that passed our screen thresholds (e-value cutoff: 10^{-50}) were further investigated as candidate anatoxin-a genes, using the same methods described above. In most samples, the anatoxin-a biosynthesis genes were located on multiple scaffolds within a genome. We successfully combined some scaffolds to get the entire gene cluster on a single scaffold by checking for identical sequences on different scaffolds and using the *de novo* assembler in Geneious v8.1.2. Then, reads from samples were mapped to the new combined scaffolds with Bowtie 2 [2] to confirm read support for the sequences on the combined scaffolds. Additionally, raw reads were mapped with Bowtie 2 [2] to the scaffold PH2015_06S_scaffold_1561, to confirm that reads from each sample mapped to the anatoxin-a gene cluster. Once all anatoxin-a genes were identified, the protein domains of the samples were compared to reference sequences using hmmscan (https://www.ebi.ac.uk/Tools/hmmer/search/hmmscan), and scaffolds were mapped to a reference anatoxin-a gene cluster from *Oscillatoria* PCC6506 (NCBI accession FJ477836.2) using Geneious v8.1.2 [7] to analyze gene synteny and sequence identity among samples and two additional reference anatoxin-a gene cluster sequences, *Anabaena* sp. 37 (NCBI accession JF803645.1) and *Cuspidothrix* (NCBI accession KM245023.1).

The relationship between the presence of the anatoxin-a gene cluster and the microbial assemblage was investigated with non-metric multidimensional scaling of Bray-Curtis dissimilarities using the R package vegan [20], and statistically tested with permutational multivariate ANOVA (PERMANOVA) [21]. Additionally, a binomial regression was used to test differences in the percent relative abundance of Burkholderiales in samples with and without the anatoxin-a gene cluster.

Anatoxin-a measurements

Anatoxin-a concentrations in Oscillatoriales mats were measured using liquid chromatography mass spectrometry (LC-MS) with Select Ion Monitoring. After a mat sample was collected for DNA extraction, ~1 g of remaining mat on the cobble was placed in a 250 mL glass jar and placed in a cooler on ice in the dark, transported to the laboratory, and stored at 4°C overnight in the dark. The next day 50-100 mL of de-ionized water was added to the sample mat, and the mat homogenized with a blender, then a 15 mL subsample transferred to a glass vial and frozen at -20°C. For anatoxin-a extraction, samples were thawed, and 3 mL sub-sample added to a glass culture tube with 3 mL of 100% MeOH (Fisher A452), then the tube was sonicated for 30 s using a probe sonicator (Sonic Dismembrator 100; Thermo Fisher Scientific, Massachusetts, USA) at ~10W power. After sonication, the tube was centrifuged (Model IEC Centra CL2; Thermo Fisher Scientific) for 5 min at 1083 rcf, and 1 mL of the supernatant was 0.2 μm filtered into an LC-MS vial. The anatoxin-a concentration in the extract was measured on an Agilent 6130 Liquid Chromatography-Mass Spectrometry system with a Cogent Diamond-Hydride column and direct-injection of 20 μL. Anatoxin-a analysis followed Cogent method 141 (MicroSolv Technology Corporation, Leland, NC, USA; http://kb.mtc-usa.com/getAttach/1114/AA-00807/No+141+Anatoxin-a+ANTX-A.pdf). Calibration was performed using certified reference

materials (National Research Council of Canada CRM ATX and Tocris anatoxin-a fumarate). Phenylalanine standards were used to check for separation between anatoxin-a and phenylalanine peaks, which can create false anatoxin-a positives in LC-MS measurements [22]. Detection limits were 0.7 parts per billion for anatoxin-a. Calibration was performed using certified reference materials with a minimum of five calibration points for each batch of samples, and analytical blanks and matrix blanks included in each run. After centrifuging and sampling the supernatant, the cyanobacterial mat in the culture tube was transferred to a weighing tin and dry weight measured after 48 hours in a drying oven at 50°C. Anatoxin-a concentrations were then calculated as ng anatoxin-a / g dry weight (DW). We also tested for a positive association between the recovery of the anatoxin-a gene cluster in samples and the detection of anatoxin-a with LC-MS using a Fisher exact test using R [24].

References

1. Holmes RM, Aminot A, Kérouel R, Hooker BA, Peterson BJ. A simple and precise method for measuring ammonium in marine and freshwater ecosystems. *Can J Fish Aquat Sci* 1999; **56**: 1801–1808.

2. Langmead B, Salzberg SL. Fast gapped-read alignment with Bowtie 2. *Nat Methods* 2012; **9**: 357–9.

3. Edgar RC. Search and clustering orders of magnitude faster than BLAST. *Bioinformatics* 2010; **26**: 2460–2461.

4. Anantharaman K, Brown CT, Hug LA, Sharon I, Castelle CJ, Probst AJ, et al. Thousands of microbial genomes shed light on interconnected biogeochemical processes in an aquifer system. *Nat Commun* 2016; **7**: 13219.

5. Hug LA, Baker BJ, Anantharaman K, Brown CT, Probst AJ, Castelle CJ, et al. A new view of the tree of life. *Nat Microbiol* 2016; **1**: 16048.

6. Edgar RC. MUSCLE: multiple sequence alignment with high accuracy and high throughput. *Nucleic Acids Res* 2004; **32**: 1792–1797.

7. Kearse M, Moir R, Wilson A, Stones-Havas S, Cheung M, Sturrock S, et al. Geneious basic: an integrated and extendable desktop software platform for the organization and analysis of sequence data. *Bioinformatics* 2012; **28**: 1647–1649.

8. Stamatakis A. RAxML version 8: a tool for phylogenetic analysis and post-analysis of large

phylogenies. *Bioinformatics* 2014; **30**: 1312–1313.

9. Parks DH, Imelfort M, Skennerton CT, Hugenholtz P, Tyson GW. CheckM: assessing the quality of microbial genomes recovered from isolates, single cells, and metagenomes. *Genome Res* 2015; **25**: 1043–55.

10. Richter M, Rossello-Mora R. Shifting the genomic gold standard for the prokaryotic species definition. *Proc Natl Acad Sci* 2009; **106**: 19126–19131.

11. Pritchard L, Glover RH, Humphris S, Elphinstone JG, Toth IK. Genomics and taxonomy in diagnostics for food security: soft-rotting enterobacterial plant pathogens. *Anal Methods* 2016; **8**: 12–24.

12. Varghese NJ, Mukherjee S, Ivanova N, Konstantinidis KT, Mavrommatis K, Kyrpides NC, et al. Microbial species delineation using whole genome sequences. *Nucleic Acids Res* 2015; **43**: 6761–6771.

13. Kim M, Oh HS, Park SC, Chun J. Towards a taxonomic coherence between average nucleotide identity and 16S rRNA gene sequence similarity for species demarcation of prokaryotes. *Int J Syst Evol Microbiol* 2014; **64**: 346–351.

14. Strunecký O, Komárek J, Johansen J, Lukešová A, Elster J. Molecular and morphological criteria for revision of the genus *Microcoleus* (Oscillatoriales, Cyanobacteria). *J Phycol* 2013; **49**: 1167–1180.

15. Sciuto K, Andreoli C, Rascio N, La Rocca N, Moro I. Polyphasic approach and typification of selected *Phormidium* strains (Cyanobacteria). *Cladistics* 2012; **28**: 357–374.

16. Criscuolo A, Gribaldo S. BMGE (Block Mapping and Gathering with Entropy): a new software for selection of phylogenetic informative regions from multiple sequence alignments. *BMC Evol Biol* 2010; **10**: 210.

17. Miller MA, Schwartz T, Pickett BE, He S, Klem EB, Scheuermann RH, et al. A RESTful API for Access to Phylogenetic Tools via the CIPRES Science Gateway. *Evol Bioinforma* 2015; **11**: EBO.S21501.

18. Haft DH, Selengut JD, White O. The TIGRFAMs database of protein families. *Nucleic Acids Res* 2003; **31**: 371–3.

19. Bergkemper F, Schöler A, Engel M, Lang F, Krüger J, Schloter M, et al. Phosphorus depletion in forest soils shapes bacterial communities towards phosphorus recycling systems. *Environ Microbiol* 2016; **18**: 1988–2000.

20. Oksanen J, Blanchet FG, Friendly M, Kindt R, Legendre P, McGlinn D, et al. Vegan: community ecology package. 2017.

21. Anderson MJ. A new method for non-parametric multivariate analysis of variance. *Austral Ecol* 2001; **26**: 32–46.

22. Furey A, Crowley J, Hamilton B, Lehane M, James KJ. Strategies to avoid the mis-identification of anatoxin-a using mass spectrometry in the forensic investigation of acute neurotoxic poisoning. *J Chromatogr A* 2005; **1082**: 91–97.

23. R Core Team. R: a language and environment for statistical computing. *R Found Stat Comput* 2018; http://www.r-project.org.
